# A Case of Laparoscopic Resection of Gastric Cancer Using Novel Laparoscopic Fluorescence Spectrum System and Near-Infrared Fluorescent Clips

**DOI:** 10.70352/scrj.cr.24-0028

**Published:** 2025-02-01

**Authors:** Shion Uemura, Yuma Ebihara, Kazuya Konishi, Satoshi Hirano

**Affiliations:** 1 Department of Surgery, Sapporo Kyoritsu Gorinbashi Hospital, Sapporo, Hokkaido, Japan; 2 Department of Gastroenterological Surgery II, Hokkaido University Faculty of Medicine, Sapporo, Hokkaido, Japan

**Keywords:** ZEOCLIP FS, Lumifinder, laparoscopic surgery

## Abstract

**INTRODUCTION:**

In laparoscopic gastrectomy, accurate marking of the lesion site is essential in determining the resection line of the stomach, owing to the lack of haptics and the direct link between negative pathological margins and prognosis. Intraoperative endoscopy may require personnel and prolong the operation time, whereas preoperative endoscopic tattooing using India ink faces problems related to the spread of ink and visibility. ZEOCLIP FS (Zeon Medical, Tokyo, Japan) is a clip made of fluorescent resin, covered by insurance since March 2019. It can be visualized from the serosal side using a near-infrared scope; however, its weak fluorescence intensity often poses viewing difficulties. Lumifinder (ADVANTEST, Tokyo, Japan) is a laparoscopic fluorescence spectrum system available for clinical use since February 2023. It can measure fluorescence intensity using a near-infrared laser and detect weak fluorescent signals. We report a case of gastric cancer in which the location of the lesion was confirmed intraoperatively using ZEOCLIP FS and Lumifinder.

**CASE PRESENTATION:**

A man in his 80s was diagnosed with gastric cancer following an examination for anemia. Two lesions were found: a 0-IIc type (cT1) at the lesser curvature of the gastric angle and a type 1 tumor (cT2) at the anterior wall of the upper gastric body. The preoperative assessment indicated no lymph node or distant metastasis. The tumor was diagnosed as cStage I and laparoscopic distal gastrectomy was planned. Two ZEOCLIP FS clips were placed on the oral side of the tumor on the anterior wall of the upper gastric body on the day before surgery. During surgery, fluorescent signals from the clips were detected using Lumifinder, enabling easy confirmation of the lesion location and determination of the gastric resection line.

**CONCLUSIONS:**

The combined use of ZEOCLIP FS and Lumifinder was a useful new method for identifying the appropriate resection line of the stomach. We plan to evaluate this method further in additional cases to enhance the detection efficacy.

## INTRODUCTION

The prognosis of early gastric cancer is good, and surgical procedures that pursue functional preservation, such as pylorus preservation and subtotal resection, are sometimes selected. Therefore, it is necessary to determine the resection line accurately. In laparoscopic gastrectomy, marking the lesion site is important to determine the resection line, which requires accuracy and safety. India ink is commonly used for intraoperative endoscopy. However, the former needs to help secure personnel and extend the operation time. The latter has issues such as the invisibility of the ink from the gastric serosa, excessive ink diffusion, and difficulty in recognizing the resection line. In a report by Yamazaki et al., India ink was not observed in 3.7% of cases, and ink was diffused in 11.1% of cases.^1)^ When clipping and India ink are used together at the same site, the center of the India ink may differ from the position of the clip.^1)^ Furthermore, the risk of perforation in colorectal cancer has been reported for India ink in colorectal cancer.^2)^

Fluorescent clips have been used to identify lesion sites more accurately than conventional clips. The ZEOCLIP FS (Zeon Medical, Tokyo, Japan) is a clip made of fluorescent resin covered by insurance in March 2019 and can be viewed from the serosal side using a near-infrared scope. However, the weak fluorescence intensity often makes viewing difficult; therefore, multiple clips around the lesion are desirable.^3)^ Kumagai et al. reported a detection rate of 75% and differences in the identification rates using near-infrared fluorescent scopes.^4)^

A novel laparoscopic fluorescence spectrum system (Lumifinder; ADVANTEST, Tokyo, Japan), available for clinical use since February 2023, measures the fluorescence intensity using a near-infrared laser, enabling the detection of even faint fluorescent signals.^5)^

Herein, we report a case of gastric cancer in which the location of the lesion was identified during surgery using ZEOCLIP FS and Lumifinder. The surgery using these devices was approved by the ethical committee in our hospital (approval number: R05-1).

## CASE PRESENTATION

**Fig. 1:**
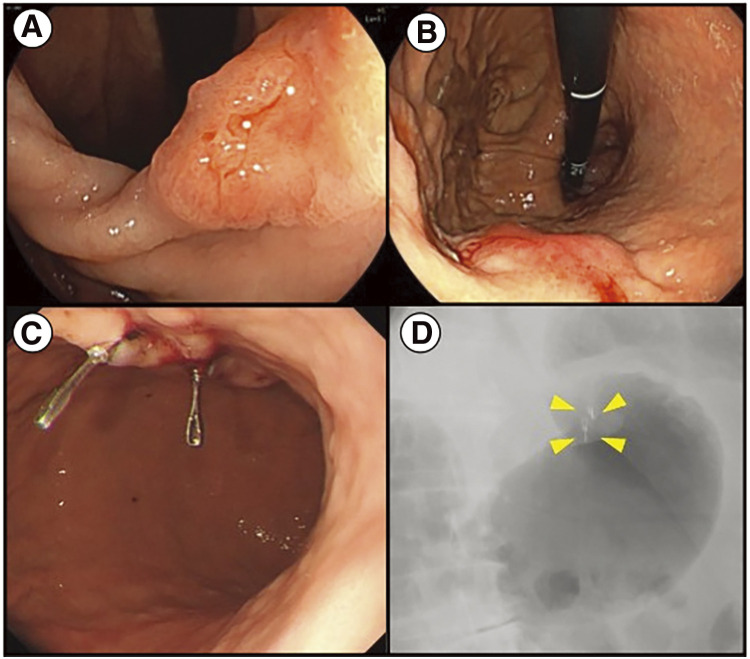
(**A**) Upper gastrointestinal endoscopy reveals a 0-IIc type tumor in the posterior wall of the lesser curvature of the gastric angle. (**B**) Upper gastrointestinal endoscopy reveals a type 1 tumor in the anterior wall of the upper body of the stomach. (**C**) Clipping on the day before surgery: Two fluorescent clips were placed on the oral side of the type 1 tumor. (**D**) Abdominal X-ray: Two fluorescent clips were identified just below the cardia (arrowheads).

A male in his 80s previously visited another hospital for systemic lupus erythematosus and was referred to our hospital for a gastrointestinal examination due to the progression of anemia. The patient presented with hypertension, dyslipidemia, hyperuricemia, chronic kidney disease, paroxysmal atrial fibrillation, and systemic lupus erythematosus. The patient had mild anemia (Hb 11.1 g/dL) and impaired renal function (Cr 1.96 mg/dL and eGFR 26 mL/min). Tumor markers were normal. Upper gastrointestinal endoscopy indicated the presence of lesions in two locations: a 0-IIc lesion (**Fig. 1A**) located slightly posterior to the lesser curvature of the gastric angle, diagnosed with a depth of invasion of cT1, and a type 1 tumor (**Fig. 1B**) located in the anterior wall of the upper gastric body, diagnosed with a depth of invasion of cT2 (both were classified as tub1 by biopsy). Chest and abdominal contrast CT showed that no thickening of the stomach wall or increased density of the surrounding adipose tissue made identifying the lesion challenging. No swelling of the surrounding lymph nodes was observed, and no findings suggested distant metastasis. The patient was diagnosed with gastric cancer in the U and M regions, classified as cT2N0M0, cStage I according to the Japanese Classification of Gastric Cancer: The 15th Edition,^6)^ and was scheduled for laparoscopic distal gastrectomy, D1+ lymph node dissection according to Gastric Cancer Treatment Guidelines 2021: Japanese Gastric Cancer Association,^7)^ and Roux-en-Y reconstruction. Considering the patient's age, the lymph node dissection was scaled down. Although the oral margin may be insufficient for type 1 tumors in the U region, a total gastrectomy was avoided in consideration of the patient’s age and underlying diseases. The day before surgery, two ZEOCLIP FS clips were placed on the oral side of the type 1 tumor in the anterior wall of the U region (**Fig. 1C**). An abdominal X-ray was then performed, confirming the presence of two clips just below the cardia (**Fig. 1D**).

**Fig. 2:**
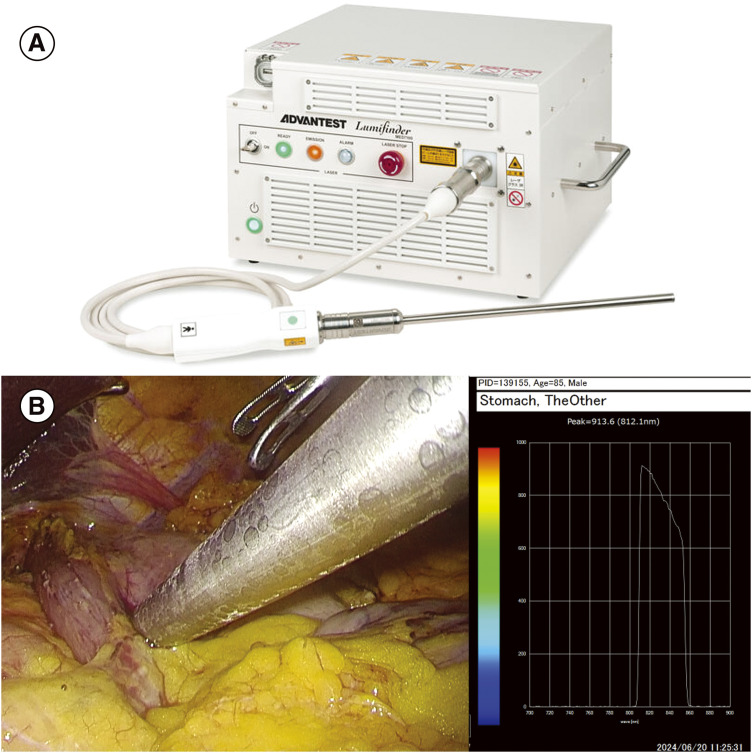
(**A**) Lumifinder device. (**B**) Searching for fluorescent clips using Lumifinder and spectral images during detection. A fluorescent signal was detected while searching the stomach wall, and a clip was identified on the oral side of the tumor.

**Fig. 3:**
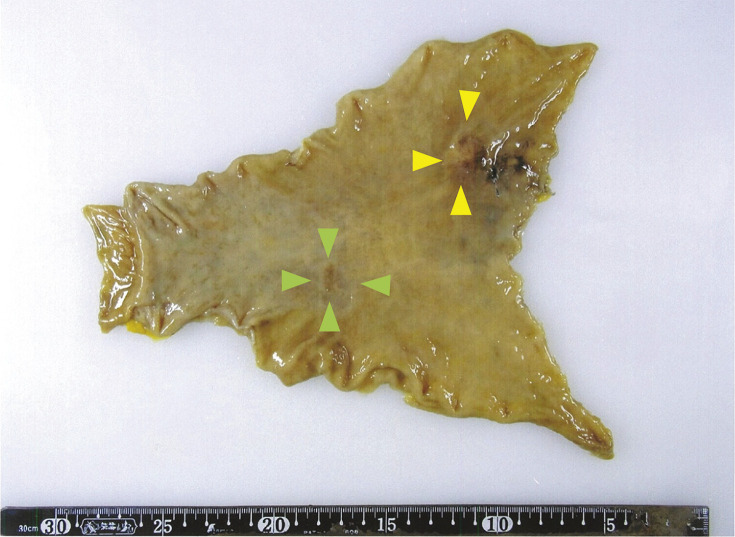
A 0-IIc + IIa lesion (green arrowheads) on the posterior wall of the lesser curvature of the gastric angle, a type 3 lesion (yellow arrowheads) on the anterior wall of the upper gastric body, and a clip on the oral side of the lesion.

The surgery was performed under epidural and general anesthesia with the patient in a supine position with his legs apart. No ascites and evident liver or peritoneal metastases were observed. The lesion in the anterior wall of the upper gastric body had invaded the serosal membrane. The lesion in the gastric angle could not be identified from the serosal membrane side. Before gastrectomy, the position of the ZEOCLIP FS clip on the oral side was easily identified using Lumifinder by detecting a wavelength of approximately 900 nm (**Fig. 2**). Roux-en-Y reconstruction was performed. The operation lasted 3 h and 46 min, with only a small amount of bleeding. The specimen was found to contain two ZEOCLIP FS clips (**Fig. 3**).

Pathological diagnosis revealed as follows:

- U area: Type 3, tub1 >tub2, pPM0, pT3N1aM0, pStageIIB^6)^
- M area: Type 0-Iic + IIa, tub1 >tub2, pDM0, pT1b1N0M0, pStageIA^6)^

On the second postoperative day, the patient developed shingles around the left shoulder. However, no complications were observed, and he was discharged on the 14th postoperative day. Considering his age and comorbidities, we decided not to administer adjuvant chemotherapy after surgery. He is currently undergoing outpatient observation.

## DISCUSSION

With recent improvements in the ability to diagnose early gastric cancer, the number of surgical procedures, such as laparoscopic distal gastrectomy and pylorus-preserving distal gastrectomy, is increasing. However, laparoscopic gastrectomy lacks haptics, making it difficult to identify lesions without serosal changes in the abdominal cavity. In addition, since a pathological margin negativity directly affects the prognosis, accurately determining the lesion is necessary.^8)^ One method exists in which the lesion site is estimated from preoperative images and then resected; however, this requires considerable experience and skill and is not universally applicable. Intraoperative endoscopy is highly reliable because the resection line can be determined while visually confirming the tumor. However, it extends the operative time and requires additional personnel. India ink is widely used because it makes it easy to identify the lesion site visually. Still, accurately identifying the lesion can be difficult if the ink does not penetrate the submucosa, the serosal membrane is not stained when observed from within the abdominal cavity, or if the amount injected is large and the staining is widespread.^1)^ In addition, spraying ink into the abdominal cavity through the serosal membrane is challenging, highlighting the need for safer and more reliable marking methods. This study evaluated a new method for determining the resection line using the ZEOCLIP FS and Lumifinder during laparoscopic gastrectomy.

The ZEOCLIP FS is a clip that uses fluorescent resin at the tip, allowing visualization from inside the lumen through the serous membrane with a near-infrared scope. Health insurance has been covered since March 2019 and can be used at facilities that have installed near-infrared scopes. Accurate identification of the lesion site is possible because it fluoresces only at the site of placement. However, because multiple clips are recommended,^3)^ challenges such as cost and difficulty of further searching when visibility during surgery is compromised.

Lumifinder is a fluorescence detection system that irradiates a near-infrared laser onto an observation site where indocyanine green has been administered in advance during laparoscopic surgery, displaying the fluorescence intensity as a number or graph. Lumifinder received clinical approval in February 2023.^9)^ The average thickness of the stomach wall is 2.55–3.09 mm,^10)^ and ZEOCLIP FS clip can detect fluorescence up to 10 mm, allowing for observation with a near-infrared scope.^4)^ However, if a clip is placed on the stomach folds, the distance between the clip and the serosa increases, making it impossible to detect.^5)^ When identifying the site of a lesion by combining the ZEOCLIP FS and Lumifinder, the terminal of Lumifinder can be pressed directly against the gastrointestinal wall, allowing detection may be possible even at longer distances than those with a near-infrared scope. Similarly, it may be possible to reduce the number of clips. In contrast, we encountered difficulty in identifying the clip with Lumifinder from the serosa side when India ink and the ZEOCLIP FS clip overlapped. Therefore, India ink should be avoided when identifying clips using Lumifinder. The combination of ZEOCLIP FS and Lumifinder to identify lesions has not been previously reported and could be helpful as a new resection line method in cases other than gastric cancer surgery. In this case, we quickly identified the position of the ZEOCLIP FS using Lumifinder. No clip loss was observed in the resected specimens. Although it was a case where securing a margin on the oral side was difficult, the pathological examination results showed negative margins, allowing for safe and accurate surgery.

## CONCLUSIONS

ZEOCLIP FS and Lumifinder are new practical methods for identifying resection lines during laparoscopic gastrectomy. Future case studies are needed to examine the safety and usefulness of these methods.

## DECLARATIONS

### Funding

All authors have no funding.

### Authors’ contributions

SU, YE, and KK performed surgeries.

SU acquired data and drafted the manuscript.

YE revised the manuscript accordingly.

All authors have read and approved the final version of this manuscript.

### Availability of data and materials

The dataset supporting the conclusions of this article is included within the article.

### Ethical approval and consent to participate

Not applicable

### Consent for publication

Informed consent was obtained from the patient for the publication of the details of this case and any accompanying images.

### Competing interests

The authors declare that they have no competing interests.

## References

[1] Yamazaki Y Kanaji S Takiguchi G Preoperative endoscopic tattooing using India ink to determine the resection margins during totally laparoscopic distal gastrectomy for gastric cancer. Surg Today 2021; 51: 111–7. 32594250 10.1007/s00595-020-02057-9

[2] Singh S Arif A Fox C Complication after pre-operative India ink tattooing in a colonic lesion. Dig Surg 2006; 23: 303. 17047331 10.1159/000096245

[3] Ryu S Okamoto A Nakashima K Usefulness of preoperative endoscopic fluorescent clip marking in laparoscopic gastrointestinal surgery. Anticancer Res 2020; 40: 6517–6523. 33109592 10.21873/anticanres.14675

[4] Kumagai K Yoshida M Ishida H Diagnostic performance of near-infrared fluorescent marking clips in laparoscopic gastrectomy. J Surg Res 2024; 300: 157–64. 38815514 10.1016/j.jss.2024.05.003

[5] Ebihara Y Li L Noji T A novel laparoscopic near-infrared fluorescence spectrum system with indocyanine green fluorescence overcomes limitations of near-infrared fluorescence image-guided surgery. J Minim Access Surg 2022; 18: 125–8. 35017402 10.4103/jmas.JMAS_165_20 PMC8830575

[6] Japanese Gastric Cancer Association. Japanese Classification of Gastric Carcinoma (October 2017 [The 15th ed]). Tokyo: Kanehara; 2017. pp. 17–26.

[7] Japanese Gastric Cancer Association. Gastric Cancer Treatment Guidelines. 6th ed. Tokyo: Kanehara; 2021. pp. 18–20.

[8] Hayami M Ohashi M Ishizuka N Oncological impact of gross proximal margin length in distal gastrectomy for gastric cancer: is the Japanese recommendation valid? Ann Surg Open 2021; 2: e036. 37638234 10.1097/AS9.0000000000000036 PMC10455052

[9] Chiba R Ebihara Y Shiiya H A novel system for analyzing indocyanine green (ICG) fluorescence spectra enables deeper lung tumor localization during thoracoscopic surgery. J Thorac Dis 2022; 14: 2943–52. 36071764 10.21037/jtd-22-244 PMC9442536

[10] Barski K Binda A Kudlicka E Gastric wall thickness and stapling in laparoscopic sleeve gastrectomy - a literature review. Wideochir Inne Tech Malo Inwazyjne 2018; 13: 122–7. 29643968 10.5114/wiitm.2018.73362 PMC5890851

